# Quadriceps Tendon Cylinder-Shaped Anterior Cruciate Ligament Reconstruction With Suspensory Fixation

**DOI:** 10.1016/j.eats.2024.103409

**Published:** 2025-01-07

**Authors:** Riccardo D’Ambrosi, Pietro Marchetti, Edna Skopljak, Federico Valli, Nicola Ursino

**Affiliations:** a IRCCS Ospedale Galeazzi–Sant’Ambrogio, Milan, Italy; b Università degli Studi di Milano, Dipartimento di Scienze Biomediche per la Salute, Milan, Italy; c Università Tor Vergata, Rome, Italy (P.M.); Department of Orthopedic Surgery, University Medical Centre, Ljubljana, Slovenia; d Faculty of Medicine, University of Ljubljana, Ljubljana, Slovenia

## Abstract

The use of autologous quadriceps tendon grafts is highly advantageous for knee reconstruction operations, encompassing both primary and revision reconstructions of the anterior cruciate ligament. Using specialized devices and avoiding the need for screws for femoral and tibial fixation, we present a minimally invasive method for quadriceps tendon cylinder-shaped anterior cruciate ligament reconstruction with suspensory fixation.

In this technical note, we aim to describe a surgical technique for anterior cruciate ligament (ACL) reconstruction using the quadriceps tendon.^1^, ^2^, ^3^, ^4^, ^5^, ^6^

## Surgical Technique

### Quadriceps Harvesting

**Fig 1:**
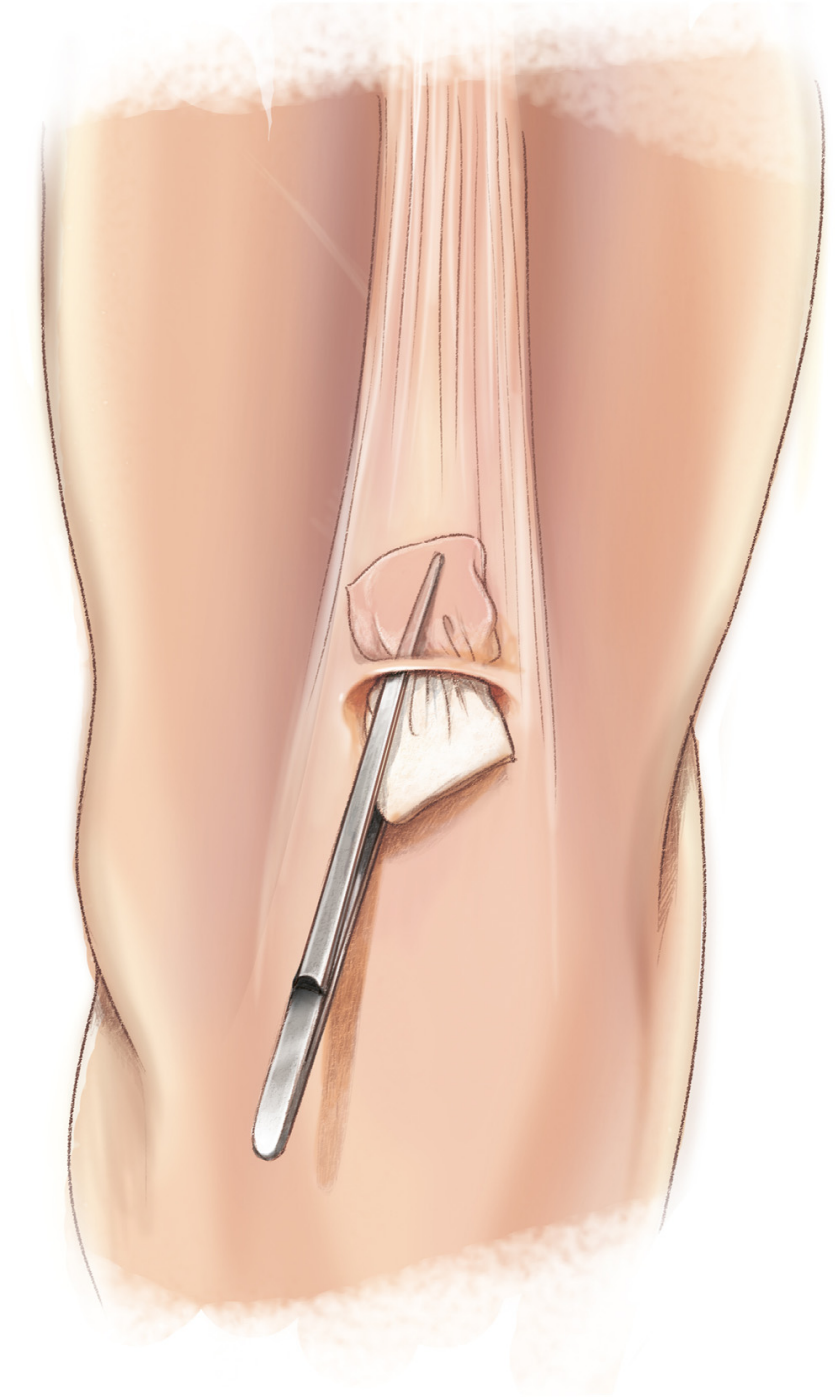
A 10 × 10–cm gauze is used to remove residual fat within the incision.

A 2.5-cm vertical skin marking is made, starting at the junction of the middle and lateral thirds of the upper pole of the patella and extending upward. A 10 × 10–cm gauze is inserted between the skin and fascia to better separate them (Fig 1). The vastus medialis is identified as a landmark so that the surgeon knows to stay lateral to it.

**Fig 2:**
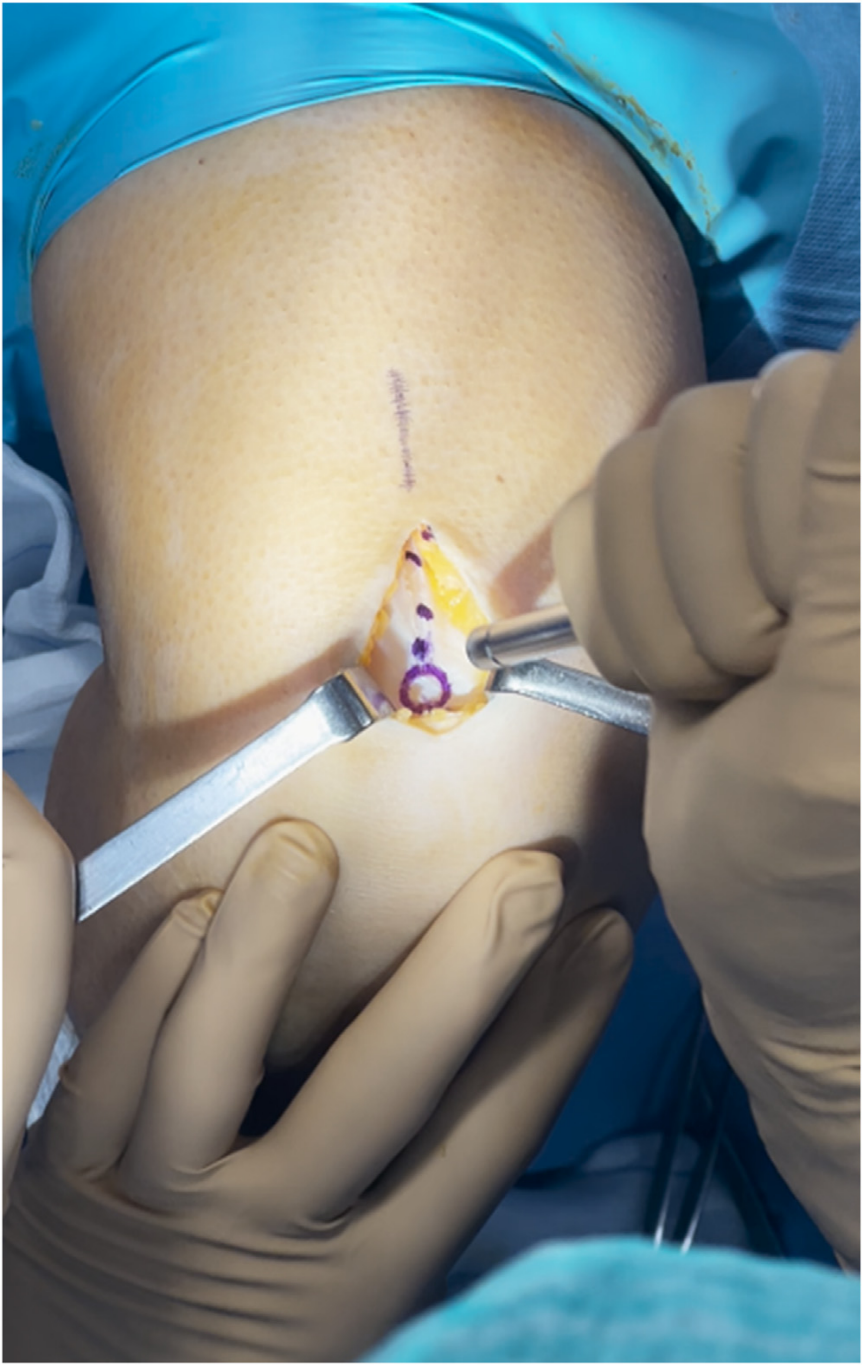
The QuadPro tendon harvester is used to make a mark and is pushed to the distal aspect of the tendon just adjacent to the patella to approximate the width of the graft.

**Fig 3:**
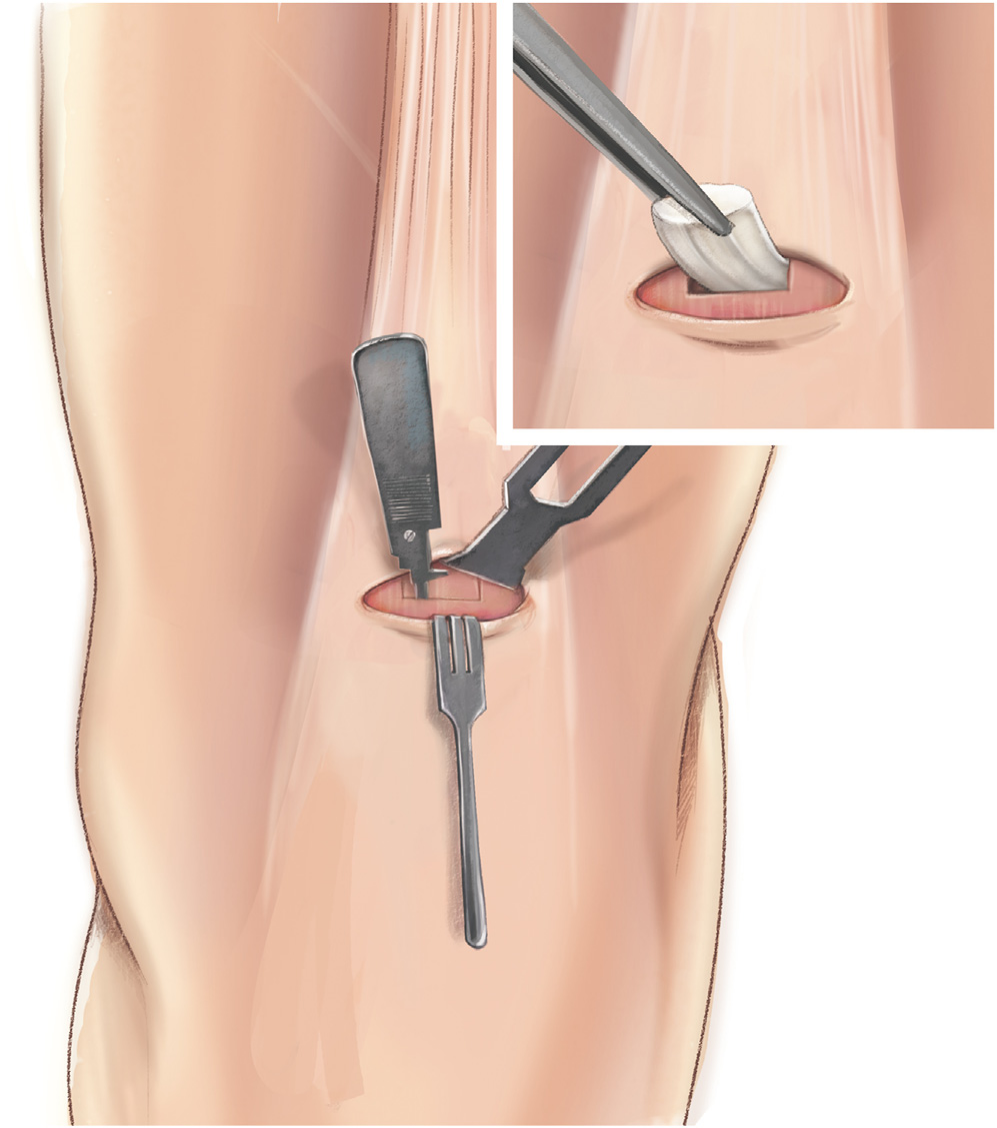
The width between incisions should be based on the desired graft diameter. Longitudinal incisions are made distally toward the patella until the bone is in contact.

**Fig 4:**
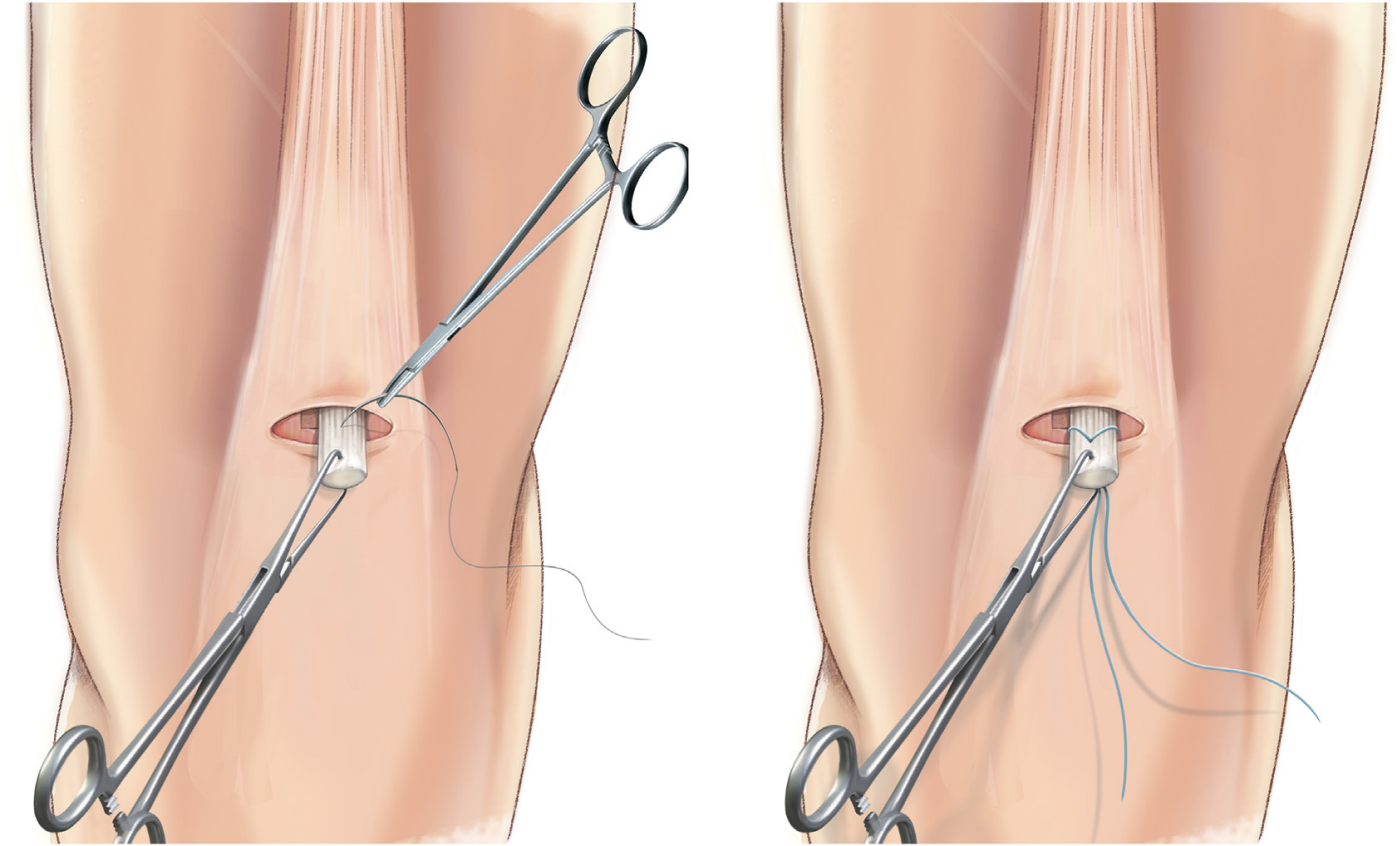
By use of a FiberLoop, the tendon is sutured 1 cm from the end of the graft. This suture functions as a tagging suture and is used to maintain tension on the graft during tendon harvesting.

**Fig 5:**
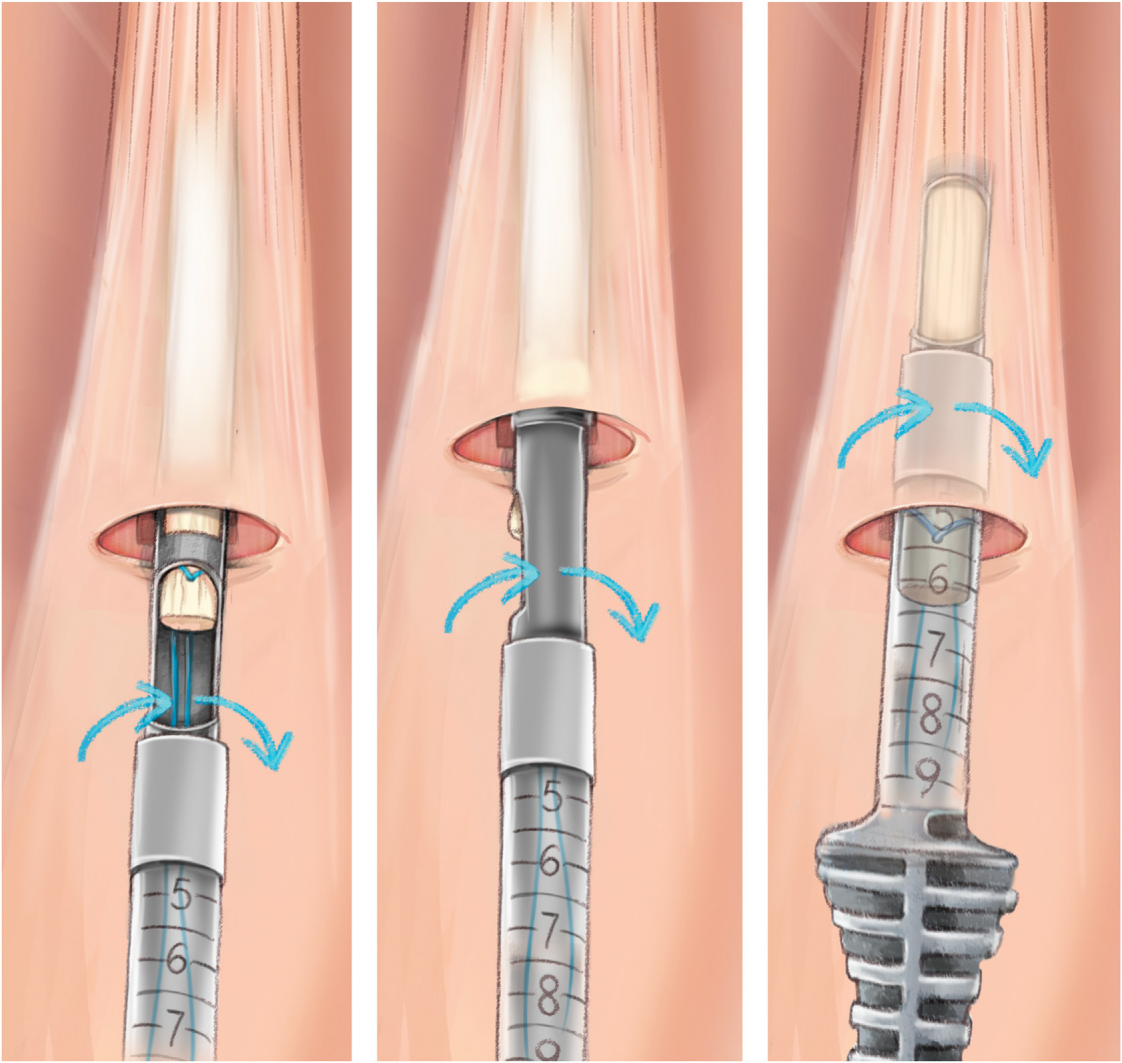
Sutures in the tendon are pulled through the cannula of the QuadPro tendon harvester. The tendon is pulled into the tip of the harvester, ensuring that the tagging suture is not cut by the harvester’s tip. Steady, gentle tension is kept on the suture, and the QuadPro harvester is rotated (blue arrows) while advancing up the tendon, directing the harvester toward the proximal mark on the skin.

**Fig 6:**
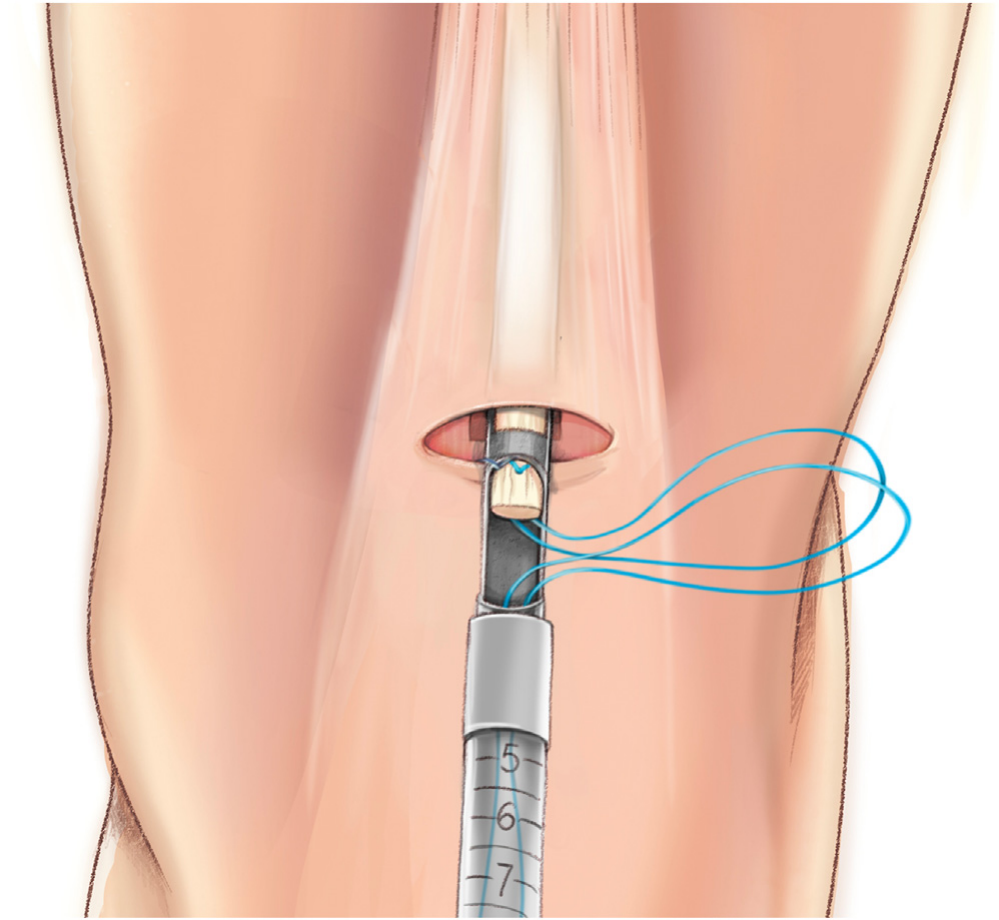
Once the desired graft length has been removed, the QuadPro tendon harvester is withdrawn from the incision, and the graft is retrieved through the graft amputation window by grasping the tagging sutures.

**Fig 7:**
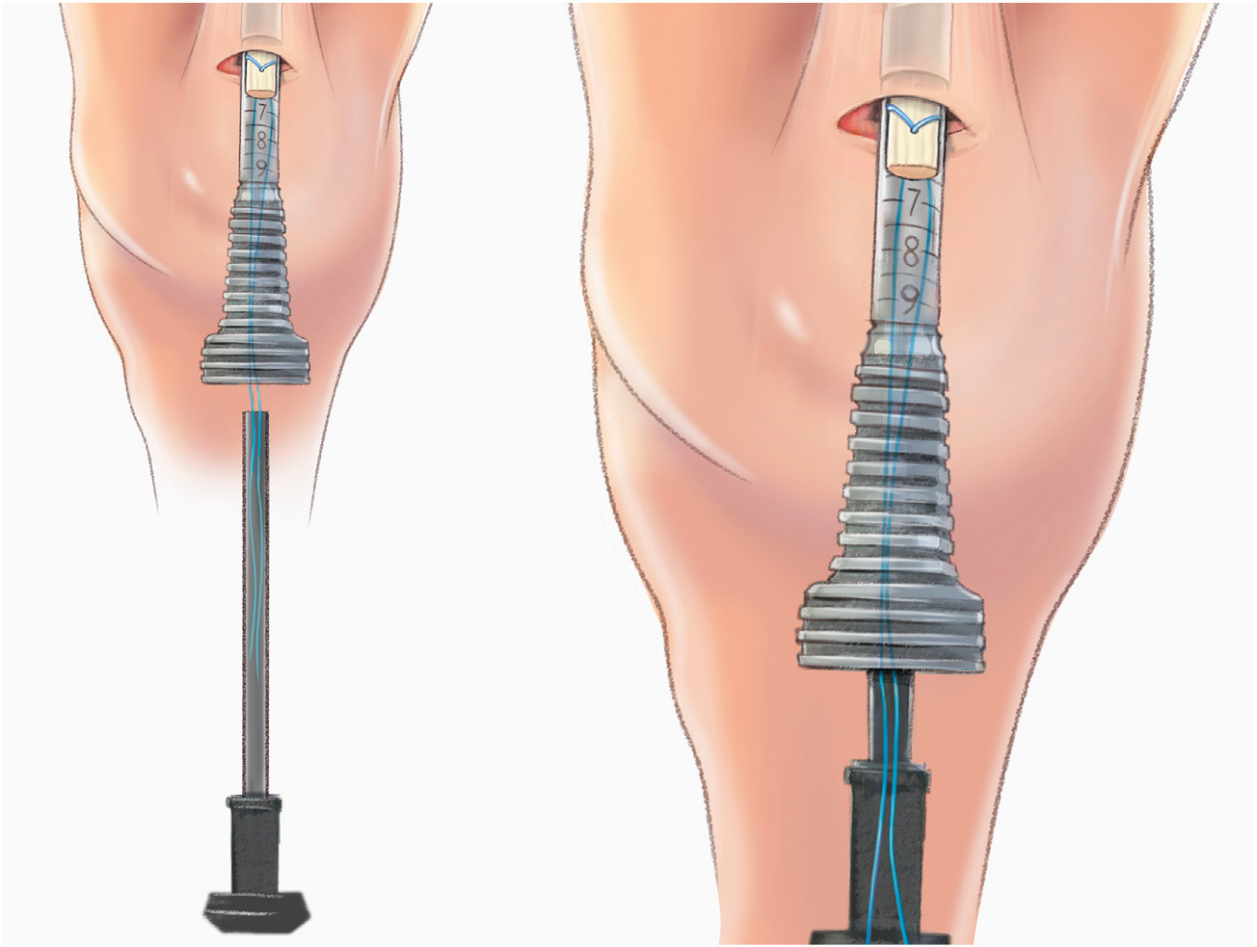
The QuadPro tendon harvester is placed back into the incision. When the appropriate graft length is reached, the push rod is inserted into the harvester. Tension is maintained on the tagging suture while the position of the harvester is maintained.

**Fig 8:**
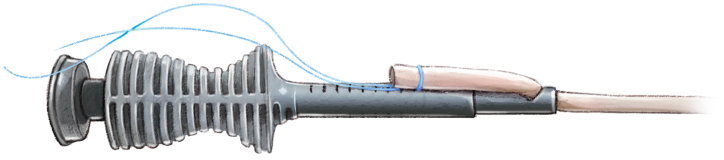
The push rod is advanced forward to amputate the graft. Deploying the push rod into the handle via a syringe-type motion allows easy amputation.

The QuadPro tendon harvester (Arthrex, Naples, FL) is used to make a mark and is pushed to the distal aspect of the tendon just adjacent to the patella to approximate the width of the transversal graft incisions (Fig 2). A scalpel is then used to make a transverse incision following the mark from the QuadPro tendon harvester, followed by 2 longitudinal incisions at the distal part of the graft. Once the free distal end of the tendon is obtained, it is grasped and lifted using Kocher forceps so that the surgeon can dissect only the superficial portion of the tendon using a scalpel and a periosteal elevator (Fig 3). The tendon is sutured 1 cm from the end of the graft from both the medial and lateral sides using a FiberLoop (Arthrex) and No. 2 Ti-Cron (Medtronic Italia, Milan, Italy) (Fig 4). The free suture ends are pulled through the cannula of the QuadPro tendon harvester. Tension is maintained with the knee flexed at 90° as the QuadPro harvester is advanced up to the tendon (Fig 5). The tendon is carefully pulled into the tip of the harvester, ensuring that the tagging suture remains intact and is not cut by the harvester’s tip. Steady, gentle tension is maintained on the suture as the QuadPro harvester is rotated, advancing up the tendon toward the previously made marking. Once the desired graft length is stripped (in this case, for the 5- to 6-cm double-suspensory system), the QuadPro tendon harvester is removed, and the graft is extracted through the graft amputation window by grasping the tagging sutures (Fig 6). The QuadPro tendon harvester is then placed back into the incision. Once the appropriate graft length is obtained, the push rod is inserted into the harvester (Fig 7). Tension is maintained on the tagging suture while the position of the harvester is maintained. The push rod is then advanced into the handle using a syringe-like forward motion to easily amputate the graft (Fig 8).

### Graft Preparation

**Fig 9:**
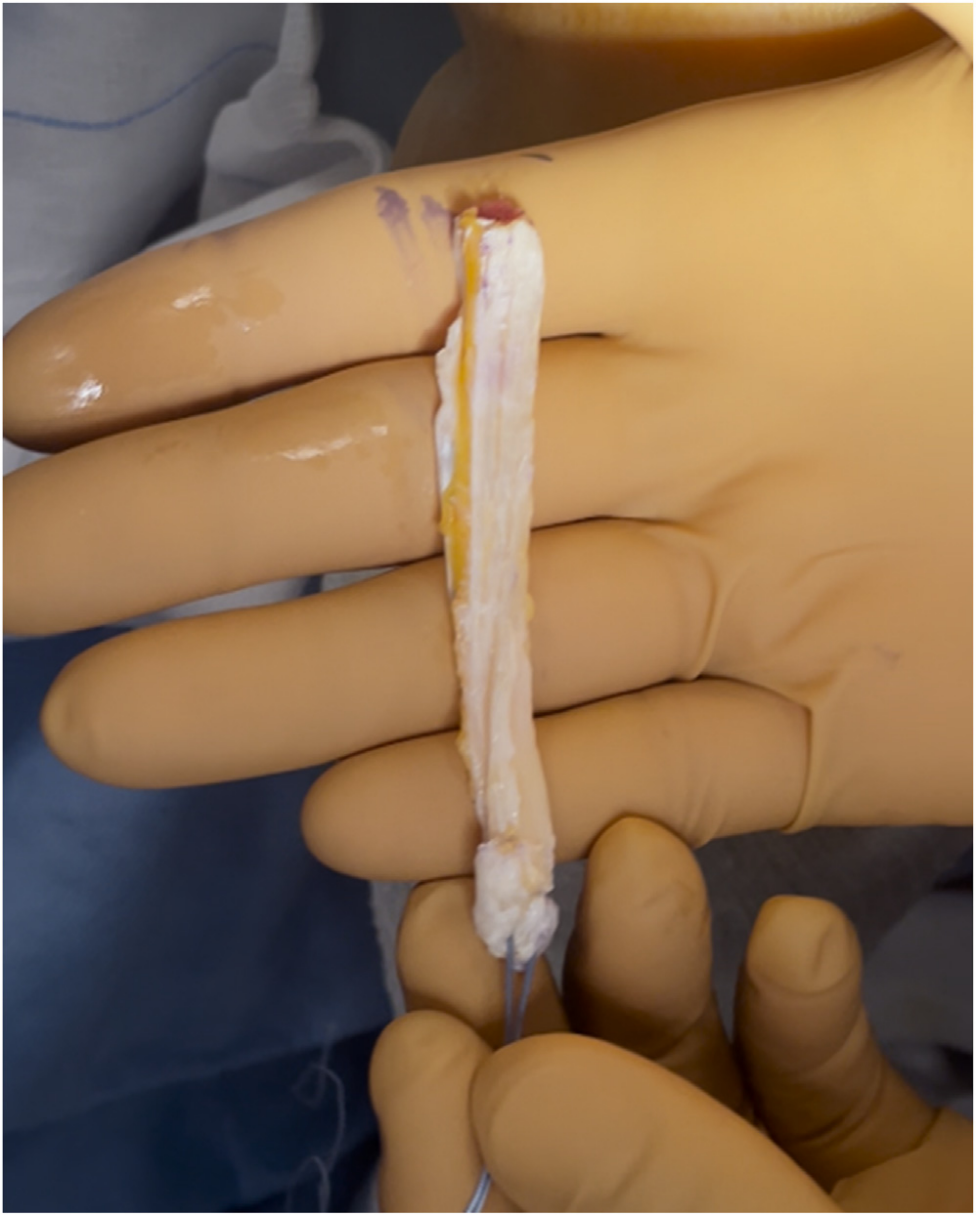
A quadriceps tendon cylinder-shaped graft is obtained.

**Fig 10:**
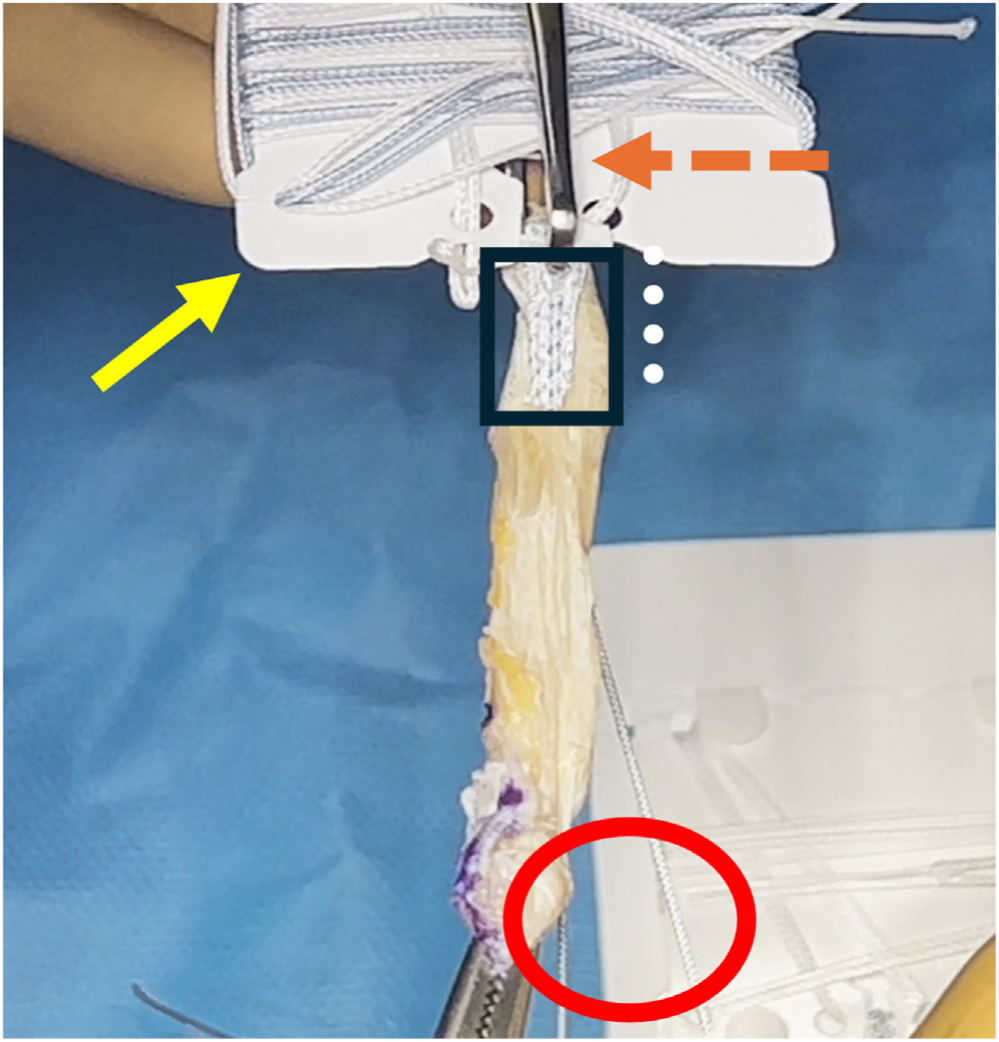
FiberTag TightRope proximal graft preparation. A TightRope suture card (yellow arrow) is loaded into the card-holding slot of the GraftClamp instrument (orange arrow). The FiberTag suture (black rectangle) is 20 mm (white dots), marking the starting point for the whipstitch. The FiberTag suture turns into the FiberLoop suture (red oval).

The total graft length is measured using a ruler, and a marker is used to indicate 20 mm from the end of the graft, marking the starting point for the whipstitch (Fig 9). The TightRope suture card (Arthrex) from the larger packaging card is removed and oriented with the FiberTag suture (Arthrex) facing the teeth of the GraftClamp instrument (Arthrex). The card is then loaded into the card-holding slot of the instrument. One tooth of the GraftClamp instrument is used to pierce the FiberTag suture (Fig 10). The GraftClamp instrument is clamped approximately 2 mm from the end of the graft, and the FiberTag suture is provisionally placed on the graft to determine the appropriate position. At this point, the position of the FiberTag suture is determined based on where the initial needle passes through the graft or where the FiberTag suture turns into the FiberLoop suture. After this initial pass through the graft, the standard SpeedWhip (Arthrex) rip-stop technique is used, working toward the TightRope implant and ensuring that the FiberTag suture is captured with each needle pass. After 2 SpeedWhip stitches on the graft, the needle is passed through the slot on the suture card, ensuring that the needle passes over the TightRope implant. The SpeedWhip rip-stop technique is then repeated in 2 additional passes, working away from the GraftClamp and ensuring that the FiberTag suture is captured with each pass.

A final pass is made at the end of the FiberTag suture. One limb of the suture just below the needle splice is cut. The suture limbs around the graft are wrapped, and the knot is tied to secure the construct using a buried knot-stack technique. Then, the suture flush is cut. The FiberTag TightRope suture card is removed from the GraftClamp instrument, the sutures are unwrapped from the suture card cleat, and the TightRope implant loops are removed from the retaining slots in the card. FiberTag TightRope proximal graft preparation is then complete.

**Fig 11:**
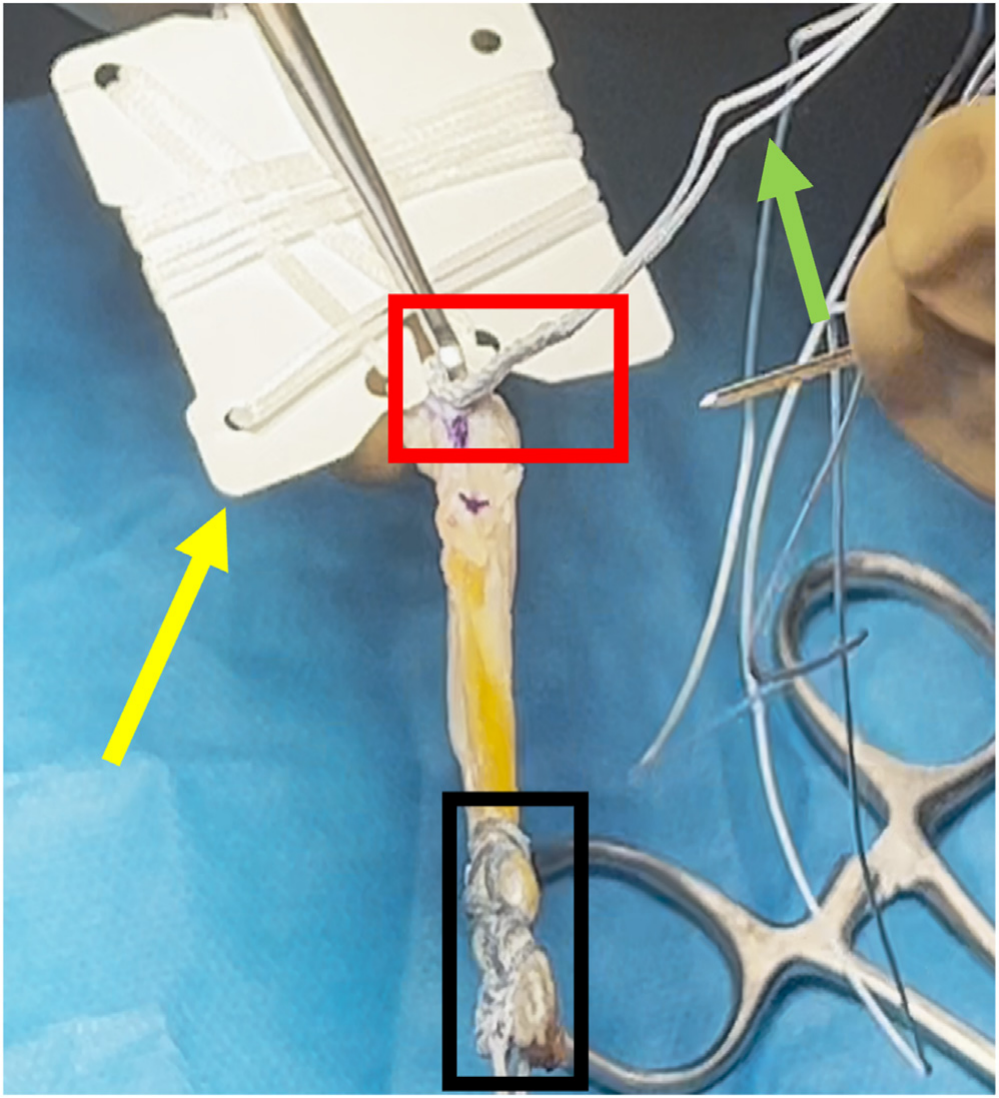
FiberTag TightRope distal graft preparation consists of the same steps as FiberTag TightRope proximal graft preparation. The black rectangle indicates the FiberTag suture; yellow arrow, TightRope suture card; red rectangle, FiberTag suture for distal graft fixation; and green arrow, FiberLoop suture free ends.

**Fig 12:**
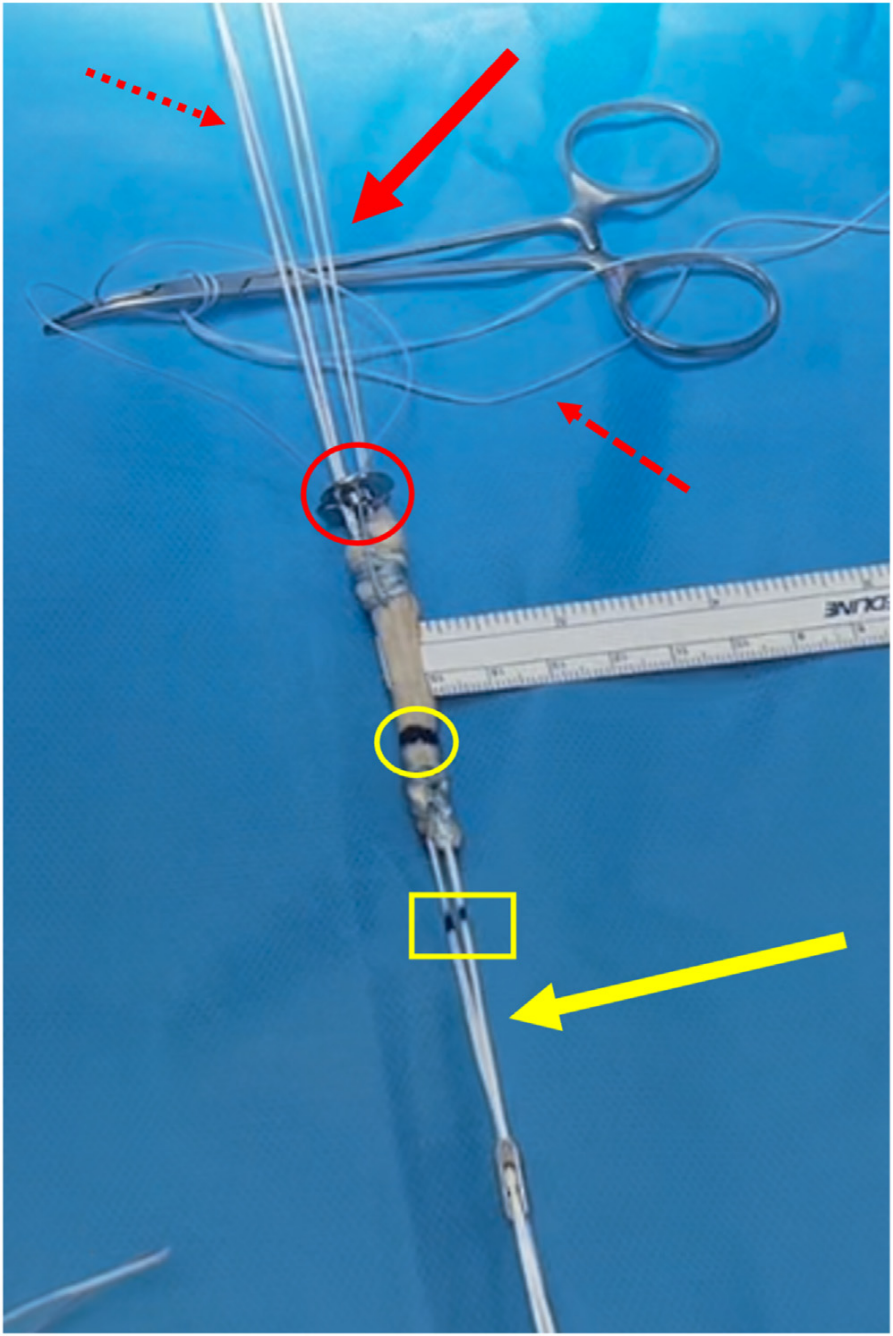
Final preparation of graft. At the distal/tibial end of the graft (solid red arrow), the white looped self-tying sliding sutures (dotted red arrow) are used to block the TightRope ABS button (red circle) on the tibial cortex. The white sutures are secured to the button through opposing slots (red dash arrow) . The blue suture is used for further safety, and the free ends are passed through small holes (red dash arrow). At the proximal/femoral end of the graft (yellow arrow), the sutures mark the length of the condyle (yellow rectangle), and the tendon is marked according to the measurement of the femoral half tunnel (yellow circle).

The distal end of the graft is prepared using the same technique described for the proximal end, given the similarity of the device (Fig 11). Once the sutures are unwrapped from the suture card, the TightRope ABS button (Arthrex) is applied to the double white sutures. Additionally, another FiberLoop is passed at the distal end of the graft; the free ends of this suture are passed through the TightRope ABS button and are used to further secure the button on the cortex (Fig 12).

### Arthroscopy Procedure

High anterolateral and anteromedial portals are established. A 4.5-mm shaver (Smith & Nephew, London, England) is used to shave the native femoral ACL footprint.

**Fig 13:**
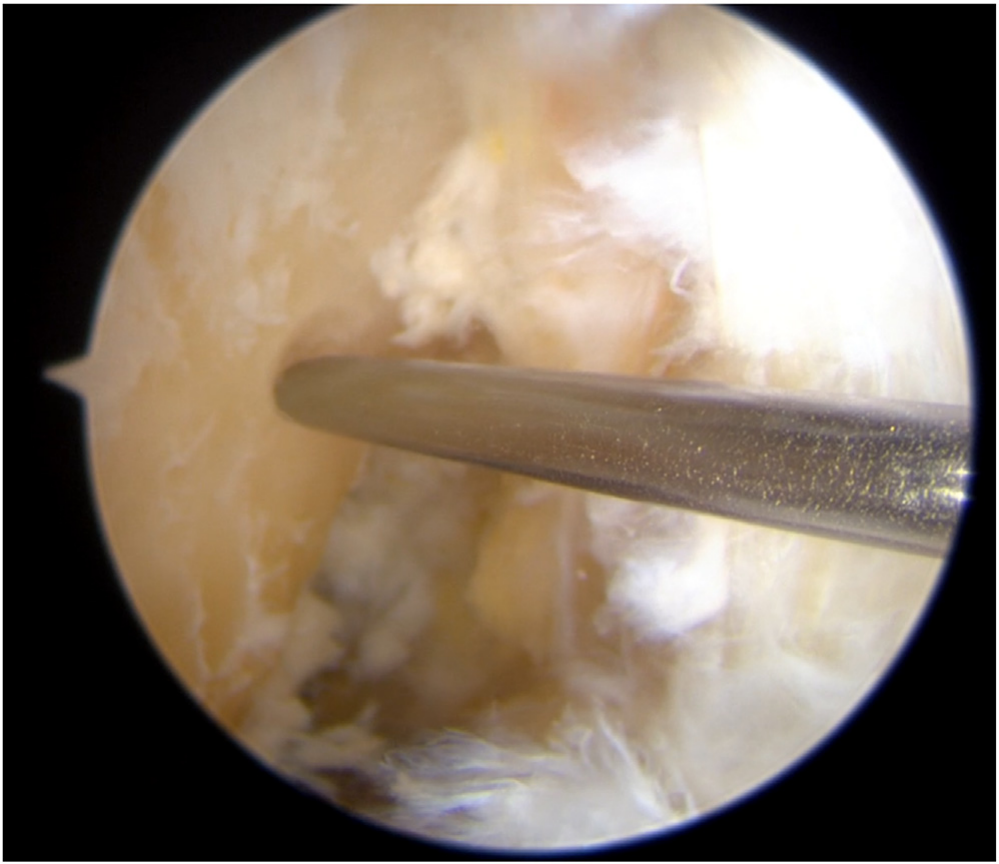
The desired femoral insertion point is identified and marked using a Chondro-Pick. A Kirschner wire is then introduced from the far medial portal to reach the desired anatomic femoral insertion. Patient supine, view from anterolateral portal. The desired femoral insertion point is established with the knee at 90°, then the the knee is flexed at 120°.

The far medial portal is made after femoral preparation. The desired insertion point is identified and marked using a Chondro-Pick (Arthrex), with the knee flexed at 90° (Fig 13). The transport wire is fed from the far medial portal to reach the desired anatomic femoral insertion with the knee flexed at 120°. The transport wire is then passed through the condyle and pierces the skin.

A 4.5-mm tunnel running the full length of the femoral condyle is drilled, guided by the transport wire, and subsequently, a half tunnel of the desired size is drilled (the half tunnel’s diameter follows the graft’s diameter, and the length of the femoral half tunnel is usually between 17 and 25 mm). The femoral tunnel is established, and a No. 2 Vicryl transport suture (Ethicon, Raritan, NJ) is pulled out of the skin near the femur, resulting in a loop at the far medial portal and free ends exiting the thigh.

**Fig 14:**
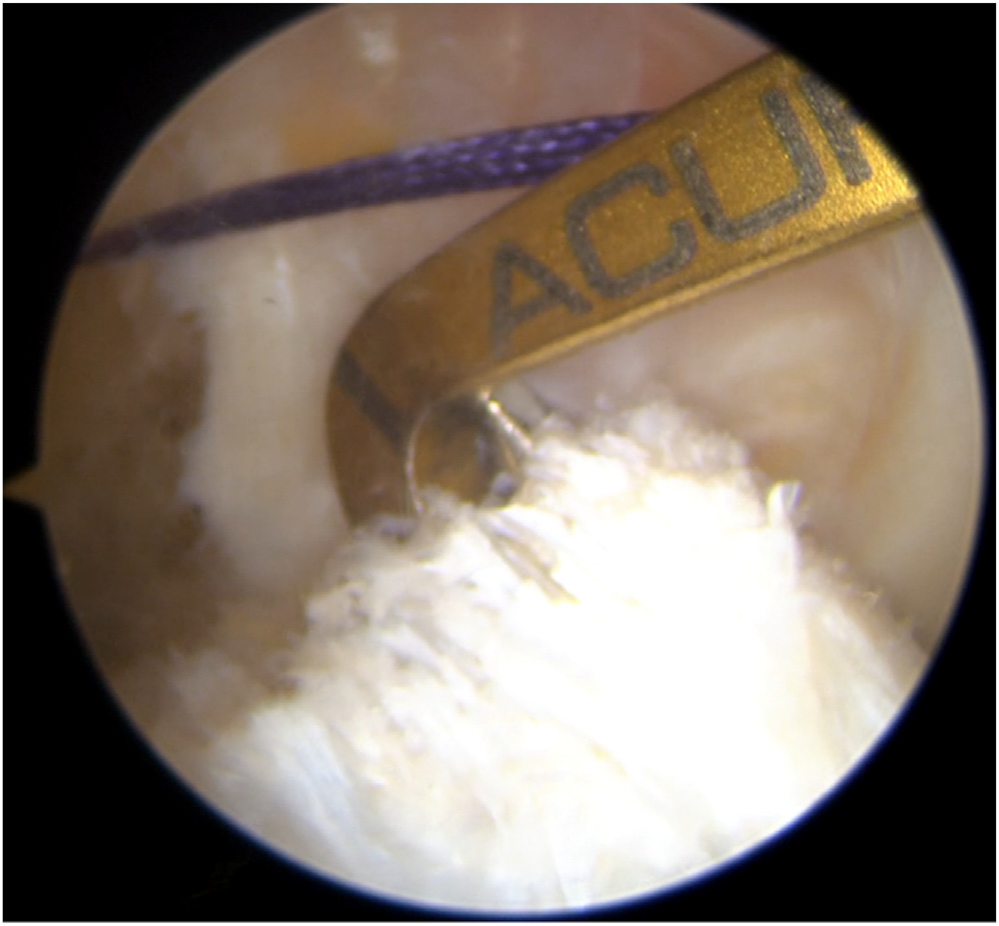
A tibial tunnel is constructed. A tibial guide and ream are used. The size of the reamer is chosen according to the size of the harvested graft. The transport suture is retrieved from the tibial tunnel. View from the anterolateral portal, with the knee flexed at 90°.

**Fig 15:**
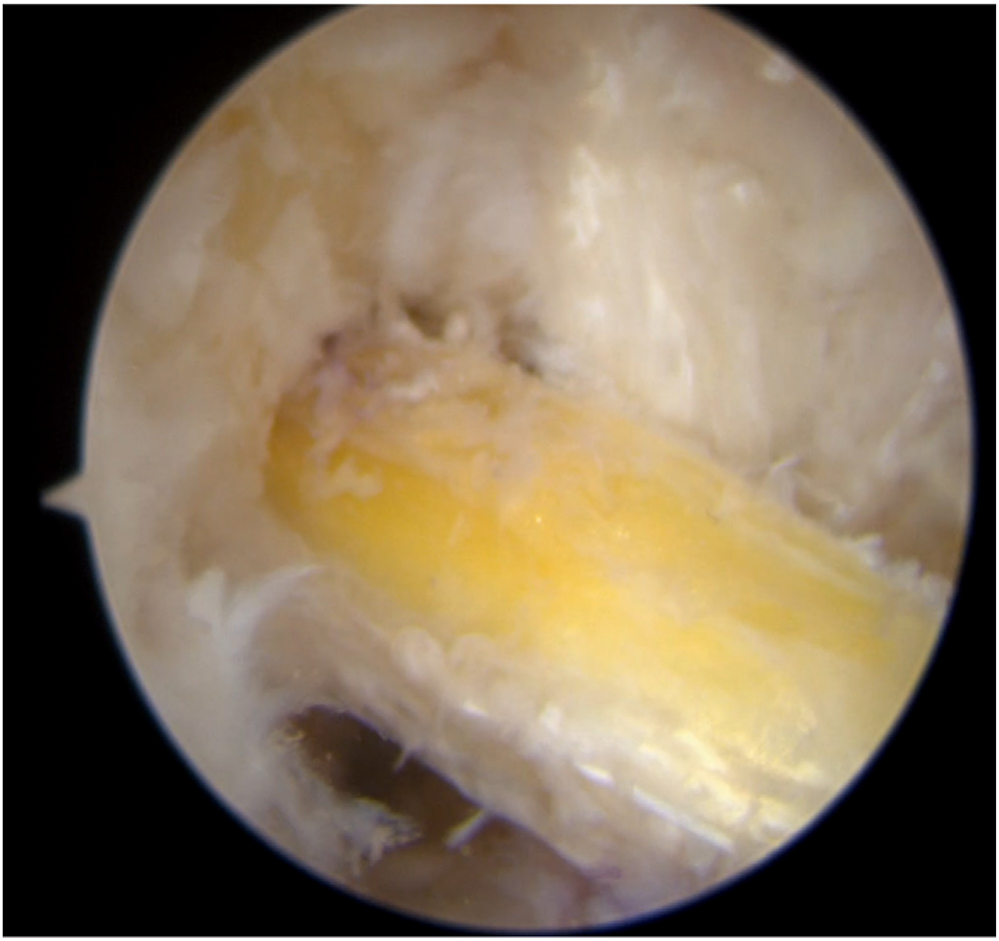
The graft is passed through the joint. View from the anterolateral portal, with the knee flexed at 90°.

The tibial guide (Smith & Nephew), set at 52.5°, is introduced through the anteromedial portal with the knee flexed at 90° (Fig 14). A guidewire is inserted, exiting the tibial plateau at the desired position. The tibial tunnel is then drilled, and the diameter is the same as that of the femoral half tunnel and graft. Accurate debridement is performed to remove all potential interference with the graft passage. The shuttle suture is retrieved from the tibial tunnel using a grasper. The graft is passed through the tibial and femoral tunnels using shuttle sutures. The graft is passed from the tibia to the femur through a skin incision, ensuring proper sliding of the graft (Fig 15).

**Fig 16:**
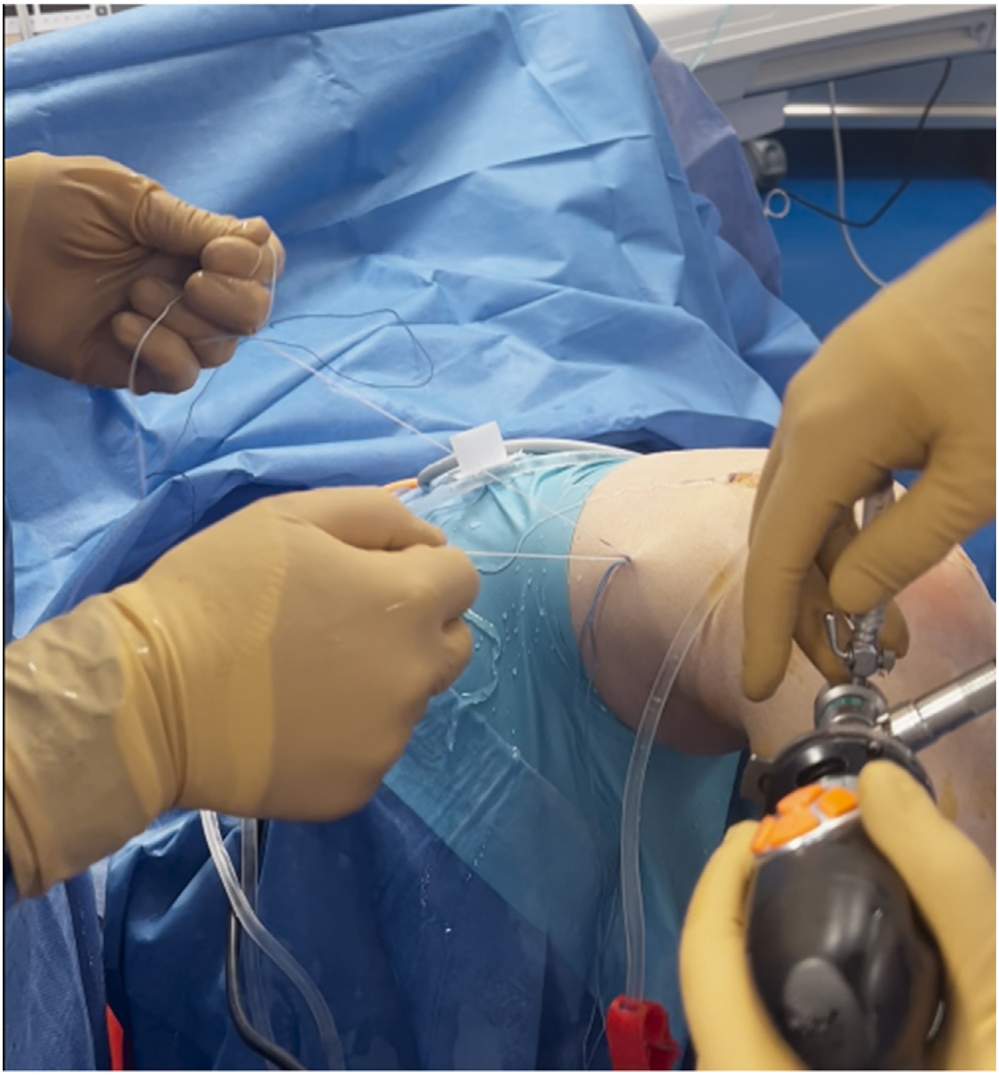
Femoral fixation. The free ends of the TightRope are pulled to ensure proper tightening of the graft in the femoral half tunnel. Knee flexed at 90°.

**Fig 17:**
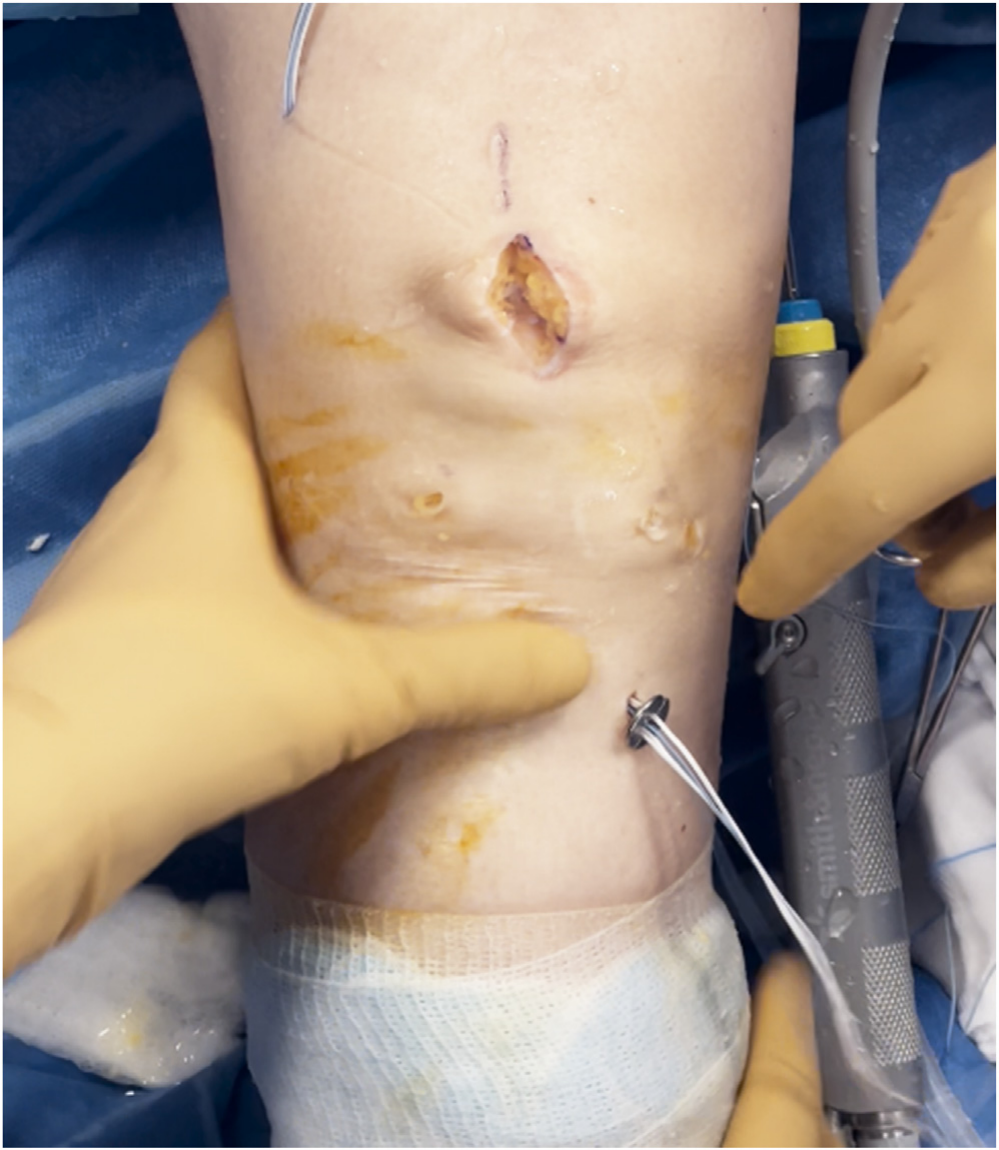
Tibial fixation. The free ends of the white sutures are pulled. This allows the loop to run back toward the bone, pulling the button on the cortex. For further safety, blue sutures are used to make another knot, ensuring proper tightening of the graft. Knee flexed at 15° to 20° and in the posterior drawer position.

The graft is first secured at the femur and then pulled with sutures on the tibia to ensure that the button is flipped and secured to the cortex (Fig 16). Knots are tied with both sutures to firmly secure the button to the cortex with the knee flexed at 15° to 20° and in the posterior drawer position (Fig 17, Table 1, Video 1).

**Table 1:** Surgical Tips and Tricks

The landmarks must be accurate for correct harvesting with the knee flexed at 90°.
A skin incision 2-2.5 cm in length must be made, starting from the upper pole of the patella at the junction of the middle third and lateral third and extending upward.
The first incision in the tendon is the medial incision, which must be made approximately 1 cm lateral to the vastus medialis.
A tendon graft approximately 1-2 cm in length must be dissected and released. It is important to size the diameter of the released graft appropriately for the preferred harvester size. A graft that is too large in diameter may not fit in the tip of the harvester.
Using controlled, quarter-turn rotations in the same direction or, alternatively, rotating the harvester back and forth during the stripping process will allow for easier advancement of the device compared with a pushing technique.
During amputation of the graft, it is important to maintain sight of the full length of the harvest to ensure the graft is not cut short.
The anteromedial portal should be checked for the complete passage of the suspensory system through the femoral tunnel.
The graft should be soaked in vancomycin.

## Discussion

This article describes an ACL reconstruction technique with cylinder-shaped soft-tissue quadriceps using a double-suspensory device. The quadriceps tendon is now recognized as a viable option for transplantation, offering a durable and mechanically advantageous graft (Table 2).

**Table 2:** Advantages and Disadvantages of Technique

Advantages
Ability to choose graft dimension
Quadriceps tendon is consistent in size (width, length, depth)
Low morbidity and good healing on harvesting site
No risk of patellar fracture
No screws
Decreased risk of neurovascular injury
Disadvantages
Quadriceps tendon graft not preferred if thickness of quadriceps tendon at 3 cm proximal to upper pole of patella in midsagittal section on magnetic resonance imaging is <7 mm
Dedicated instruments needed for tendon harvesting
Technique not suggested in patients with previous anterior cruciate ligament reconstruction with bone–patellar tendon–bone graft
Risk of penetrating suprapatellar pouch, complicating subsequent arthroscopy, especially when full-thickness graft is harvested

Recently, Runer et al.^7^ showed that harvesting an additional patellar bone block in quadriceps tendon ACL reconstruction does not seem to affect postoperative patient-reported outcomes or ACL revision or contralateral ACL reconstruction rates. Furthermore, a recent study investigated the healing of the quadriceps tendon donor site after partial-thickness graft harvesting, and the results suggested that the quadriceps tendon has a high capacity for healing after graft harvesting 6 months after surgery.^8^

Finally, for the creation of the tunnels, a far medial portal is used to ensure an anatomic femoral position. Recently, Feng et al.^9^ revealed that the anteromedial portal technique is superior to the standard transtibial technique in terms of knee stability and functional recovery.

## Disclosures

All authors (R.D., P.M., E.S., F.V., N.U.) declare that they have no known competing financial interests or personal relationships that could have appeared to influence the work reported in this paper.

## Supplementary Data

Video 1 Key steps of technique. The video is divided into 3 key steps: (1) harvesting the quadriceps using dedicated instruments to obtain a graft of the desired size; (2) measuring the tendon and mixing it with saline solution and 1 g of vancomycin for preparation of a double-suspensory system; and (3) creating the anteromedial portal with the knee flexed at 120° and creating the femoral tunnel at the desired length and size. The tibial anterior cruciate ligament (ACL) guide is set at 52° to 53° and then introduced through the anteromedial portal. The tibial tunnel is established. The transport wire is retrieved from the tibial tunnel with a grasper. The quadriceps graft is fixed with a double-suspensory system both on the tibia and on the femur. The patient is positioned supine on the operating table, with lateral support placed at the level of the thigh and a foot roll positioned to stabilize the leg in 90° of knee flexion. During arthroscopy, the view is from the anterolateral portal.
